# Supplementary material for: Deep phylogeny of cancer drivers and compensatory mutations
Source: Commun Biol. 2020 Oct 2;3:551. doi: 10.1038/s42003-020-01276-7 (PMC7532533; doi:10.1038/s42003-020-01276-7)
Supplement: Supplementary file 1 — Supplementary Information [file 42003_2020_1276_MOESM1_ESM.pdf]

Deep Phylogeny of Cancer Drivers and Compensatory Mutations

Nash D. Rochman[1], Yuri I. Wolf[1], and Eugene V. Koonin[1,*]

[1]National Center for Biotechnology Information, National Library of Medicine, Bethesda, MD 20894

For correspondence: koonin@ncbi.nlm.nih.gov

Keywords: cancer driver gene, cancer driver mutation, compensatory mutation, deep phylogeny, epistasis within proteins

Supplementary Figures

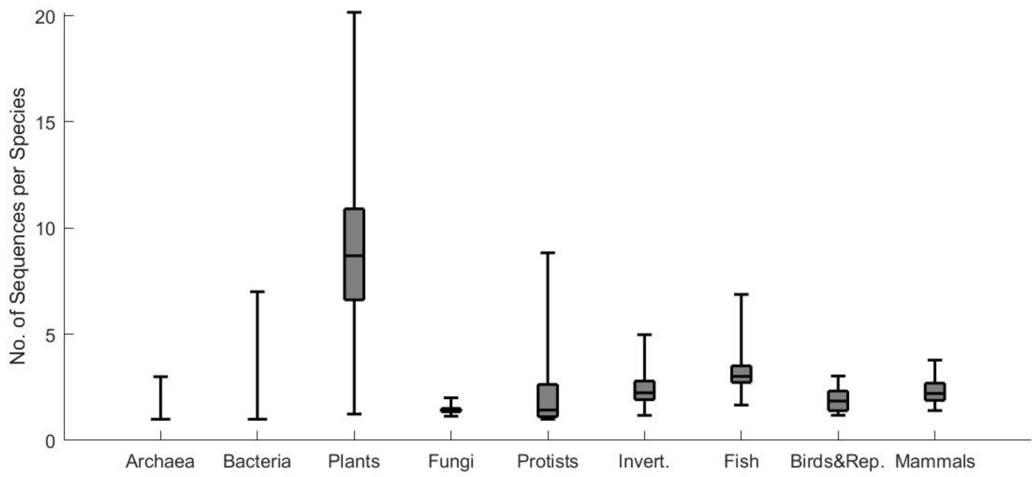

Supplementary Figure 1. Number of Sequences Per Species Per MSA Box plot of major taxonomic groups.

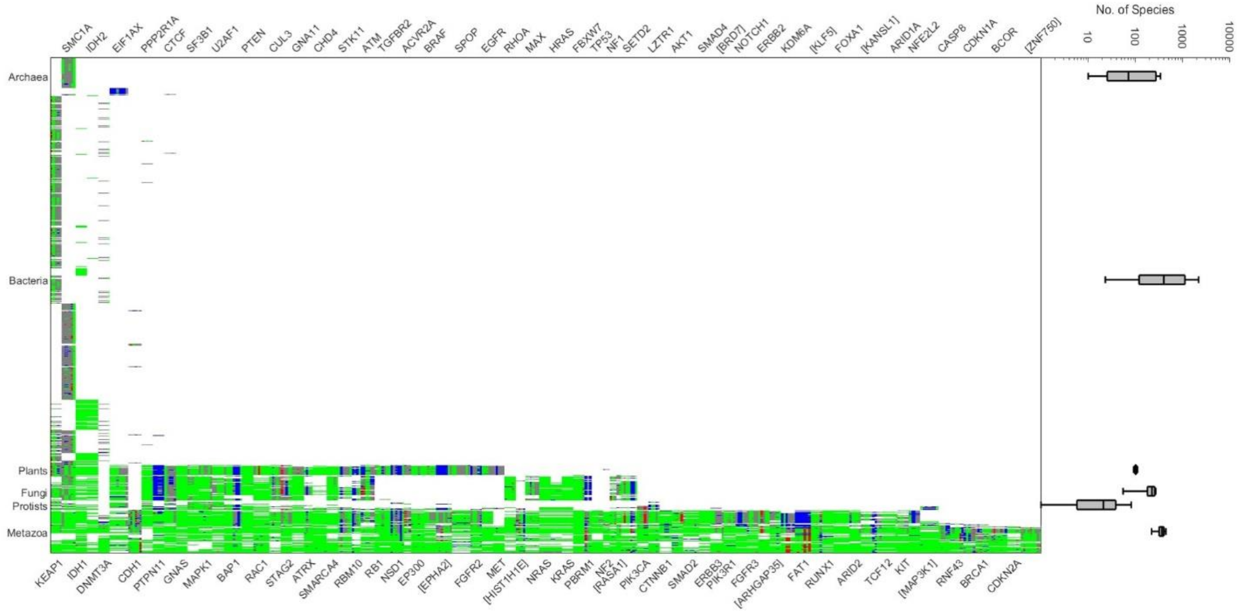

Supplementary Figure 2. Cancer Driver Phylogeny Through Prokaryotes Summary of driver phylogeny. Each row represents one species, each column one driver. Sites harboring multiple drivers appear multiple times. Colors correspond to mode residues over all sequences from each species in each site: white=absent from MSA, blue=gap, green=human reference residue, red=driver, gray=any other residue. Species are ordered by taxonomy, and within labelled clades by appearance within # of MSA. Sites are ordered by harboring gene phylogenetic depth. Rows are followed by box plots of number of species within each clade observed across MSA where that clade is represented. Whiskers are at 2/98%. For a focused view on Eukaryotes see Fig. 1F/H.

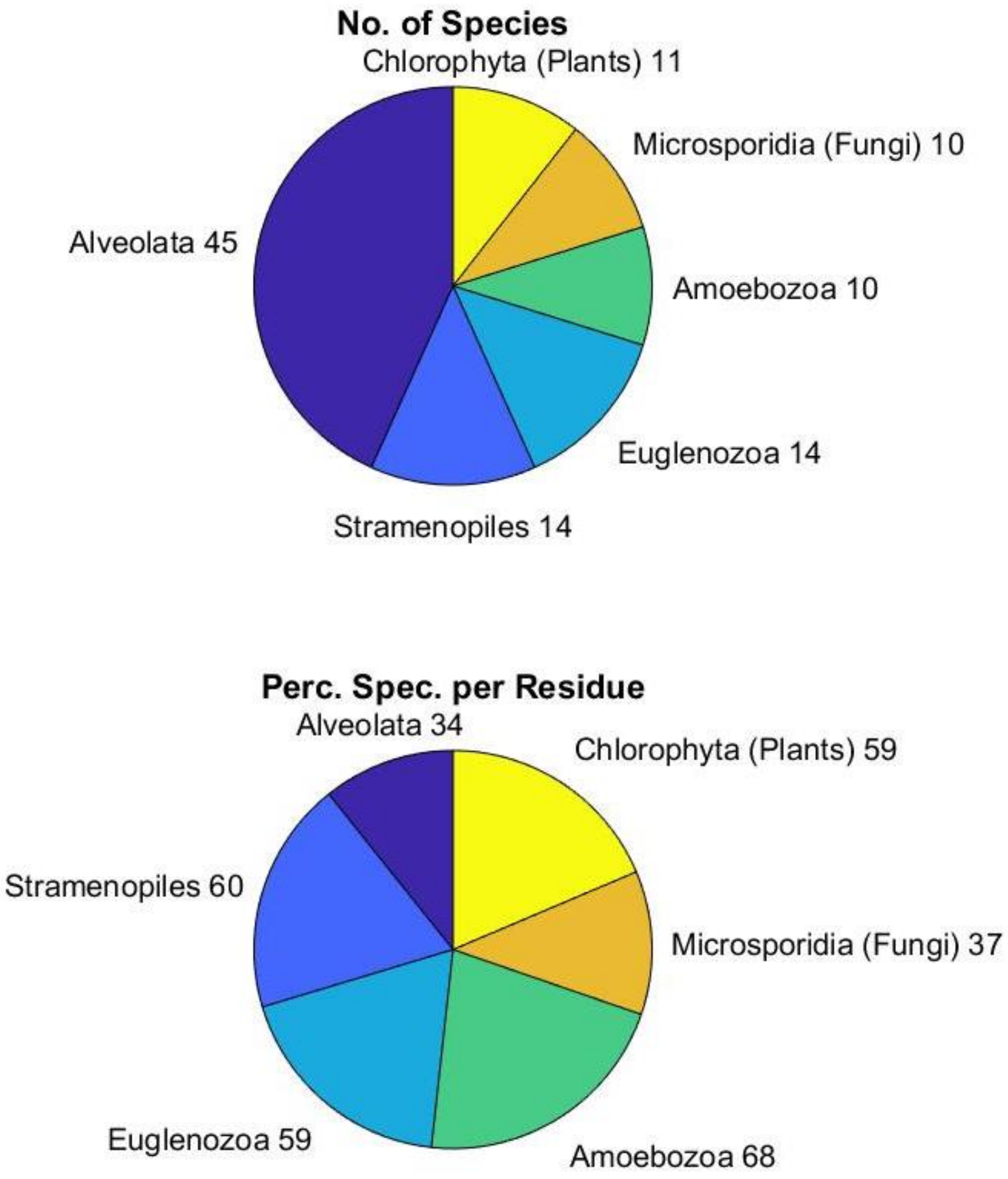

Supplementary Figure 3. Available Protist Phylogeny Few protists appear across all MSA. Most are in Alveolata. Members of Alveolata are particularly unevenly distributed across the MSA with only 34% of total species present in any MSA found in any given MSA (Percent Species per Residue).

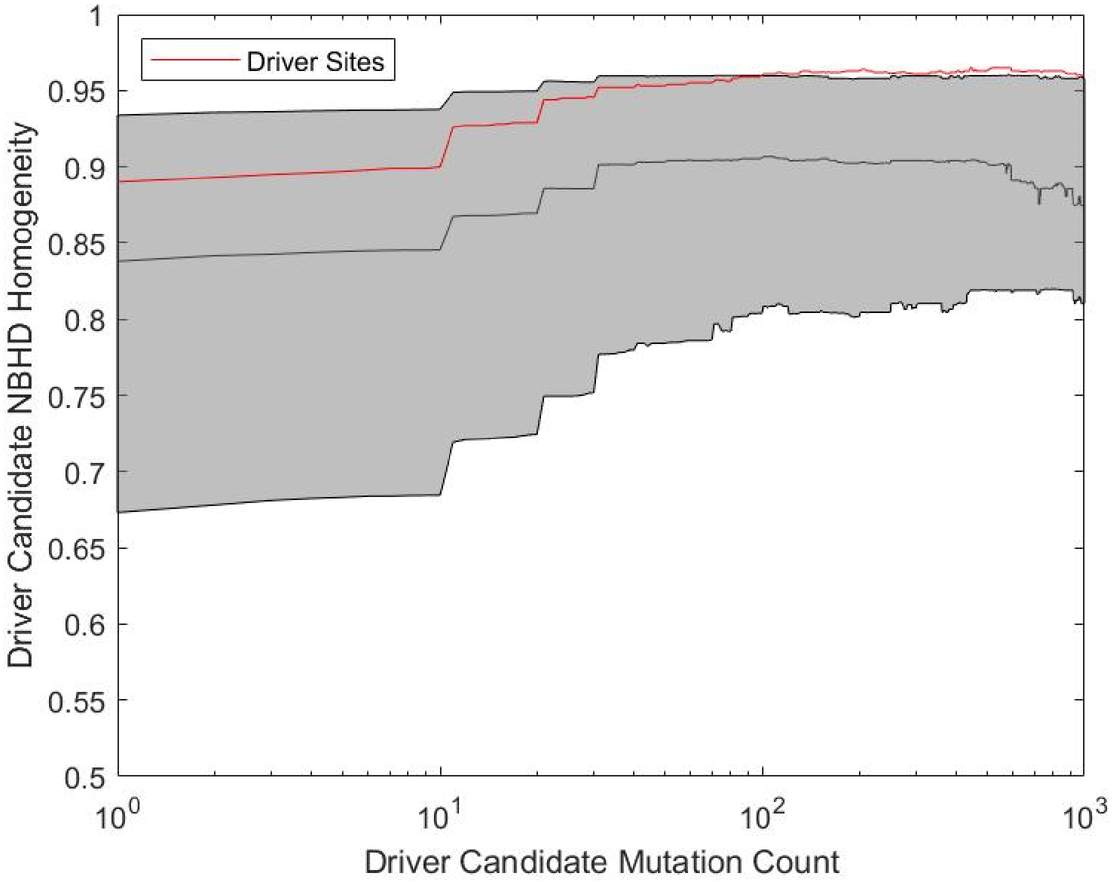

Supplementary Figure 4. Vertebrate Homogeneity Binned By Mutation Frequency

Median vertebrate homogeneity of COSMIC mutation nbhds (+/- 3sites) and sites logarithmically binned by mutation count (number of tumors). 25th-75th percentiles shaded.

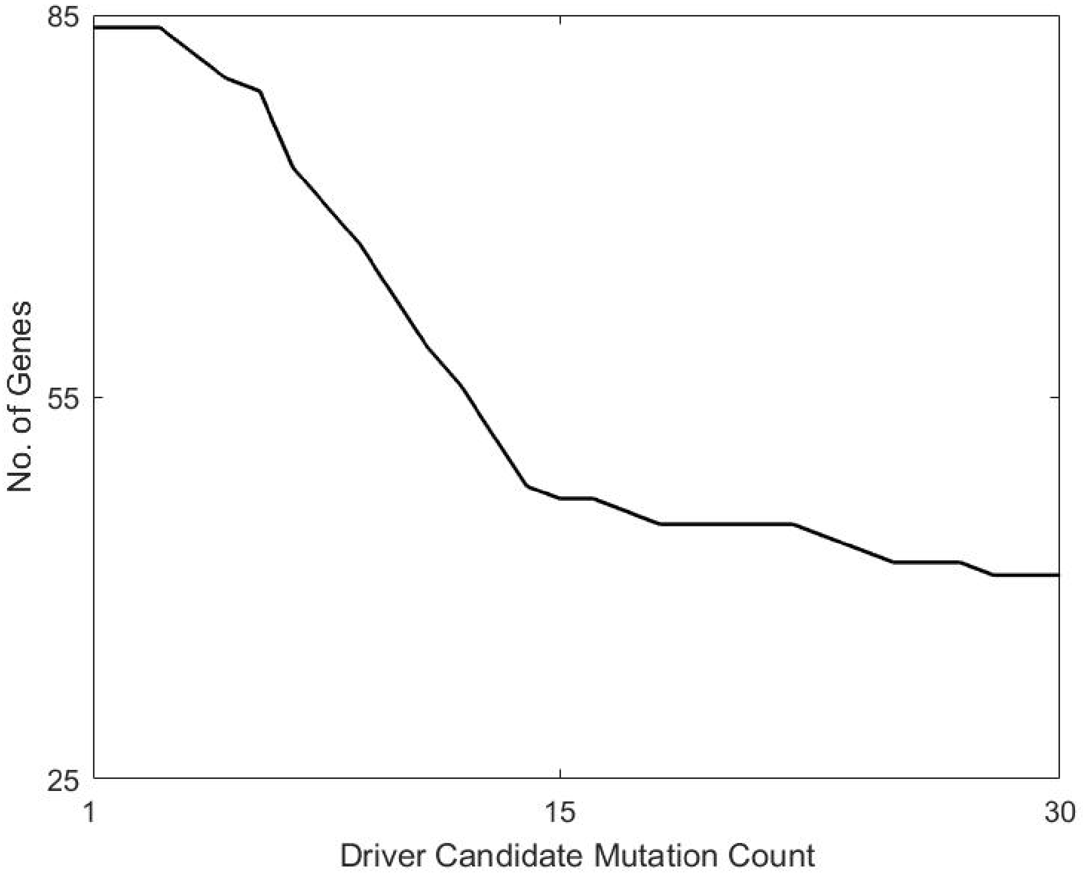

Supplementary Figure 5. Number of Drivers with Given Mutation Frequency

#Driver genes (84 total) with at least 1 missense mutation of specified cosmic mutation frequency.

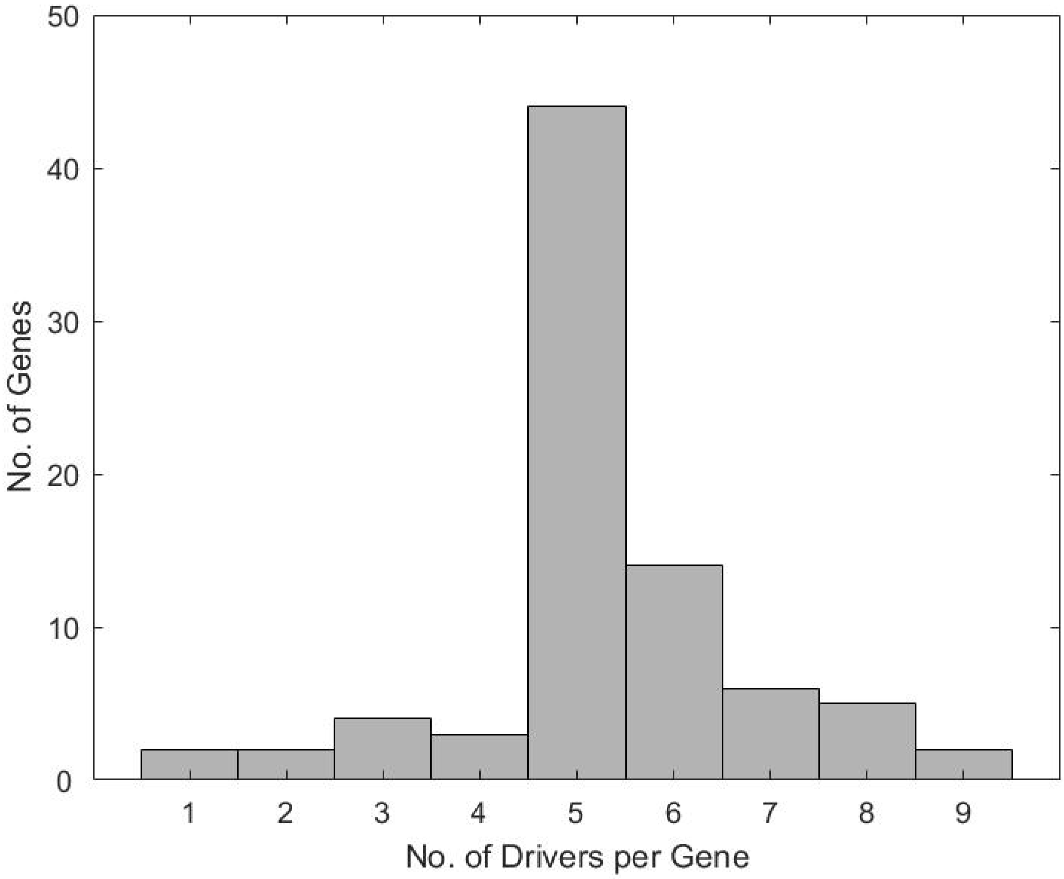

Supplementary Figure 6. Number of Drivers Designated per Gene

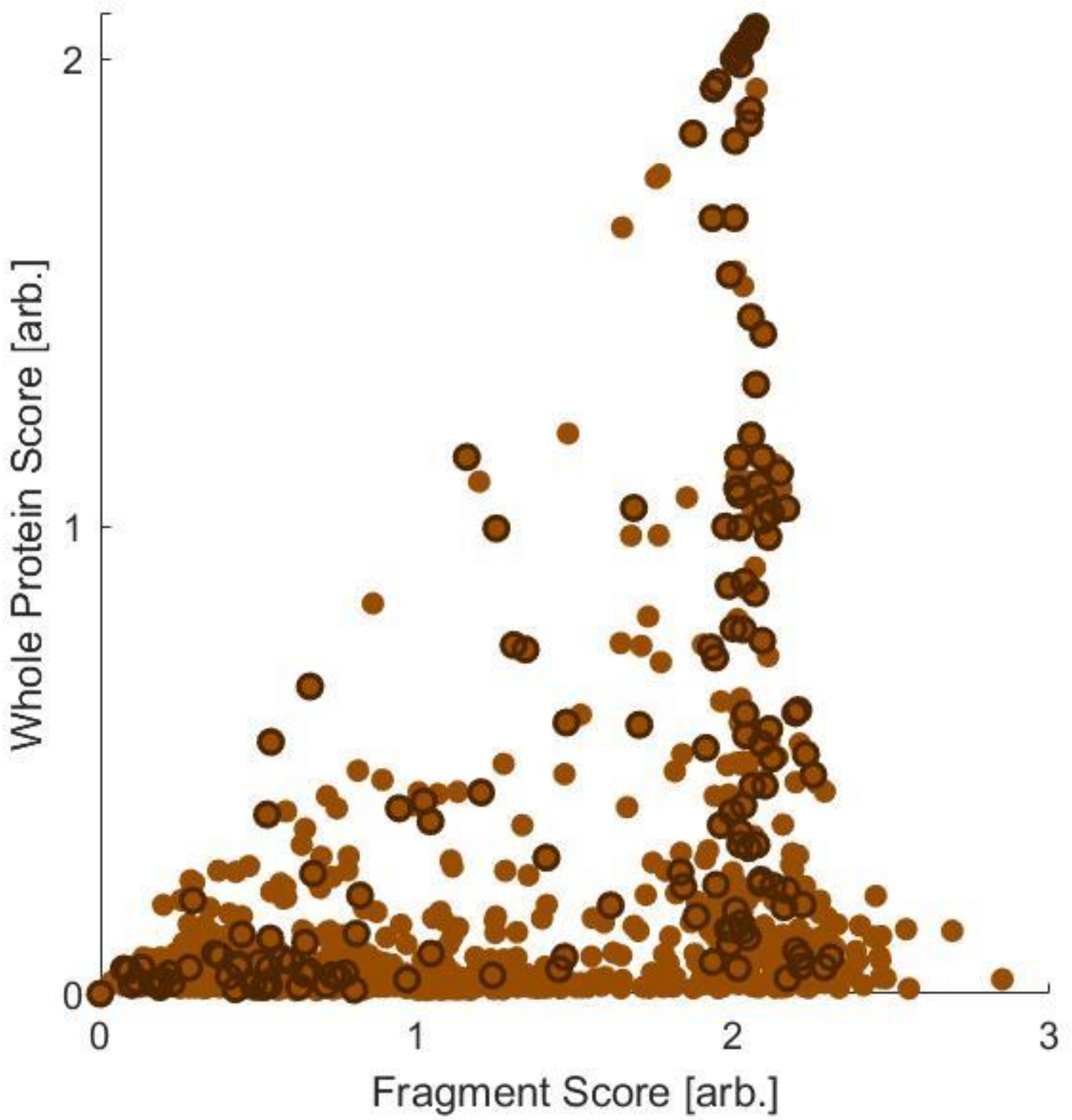

Supplementary Figure 7. Maximum Identity Score Against PDB Structures All sites in driver genes with driver sites outlined in black. Global Score vs. driver-containing Local Score. Global Score ≤ Local Score, see Methods; Fig. S8 for details.

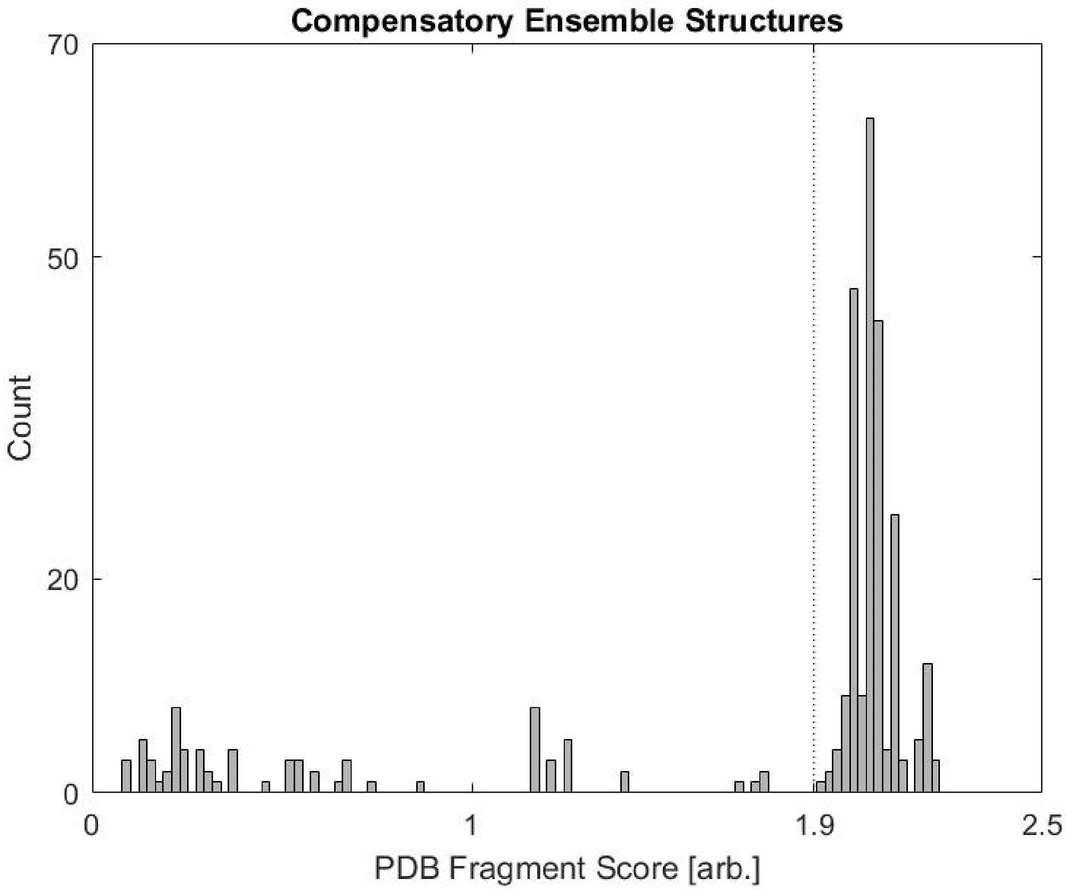

Supplementary Figure 8. Distribution of Local Scores for PDB Structures Encompassing Drivers and Compensatory Ensembles Vertical line at threshold score of 1.9.

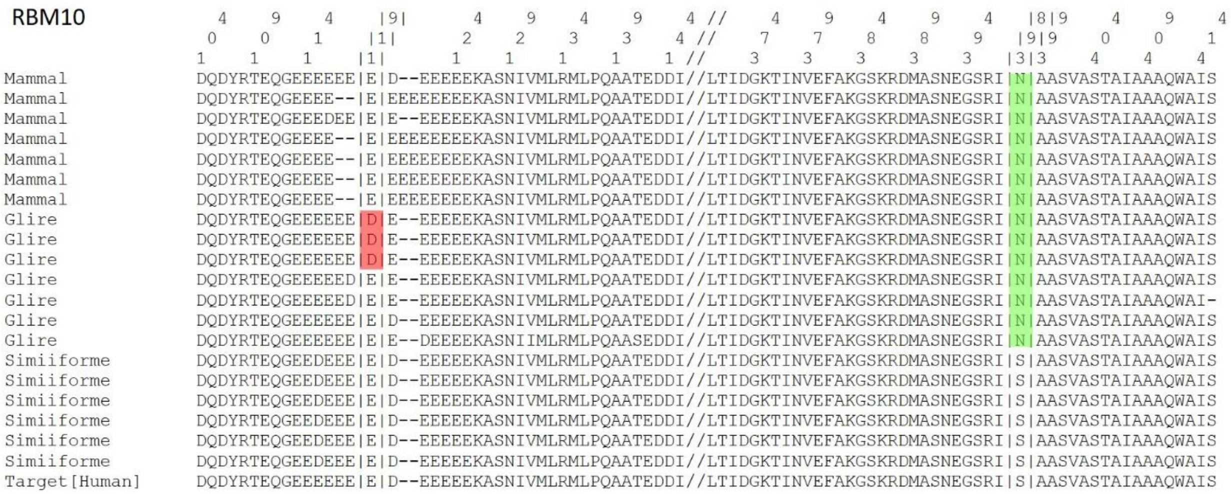

Supplementary Figure 9. Reduced MSA for driver gene *RBM10* Driver E119D and compensator S398N highlighted. The driver neighborhood in the MSA is almost exclusively composed of gaps outside of mammalian sequences. Within mammals there are multiple isoforms of *RBM10* about half of which have gaps in the driver neighborhood as well. The compensator residue 398N is ancestral transitioning to the human reference residue 398S at siimiformes and the driver is relatively common among mammals, approaching 15% among glires suggesting the driver residue may only be deleterious among higher primates due to the absence of the compensator S398N.

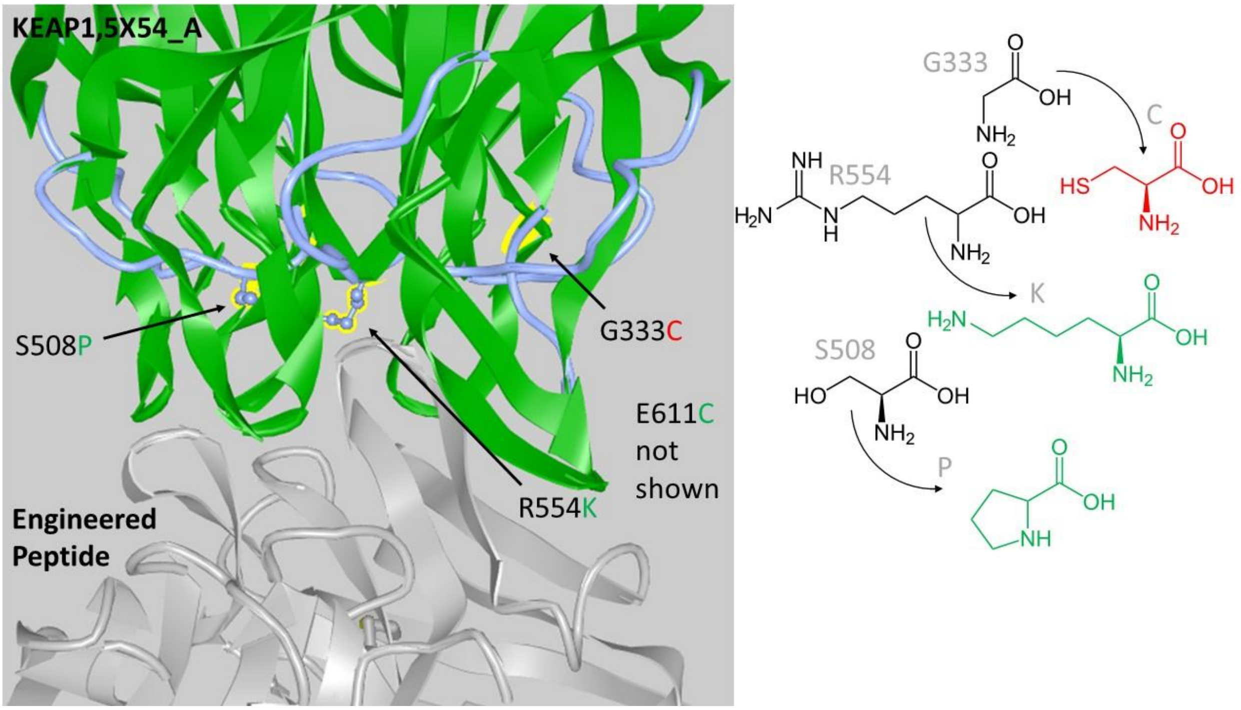

Supplementary Figure 10. Detailed View of *KEAP1* Structure

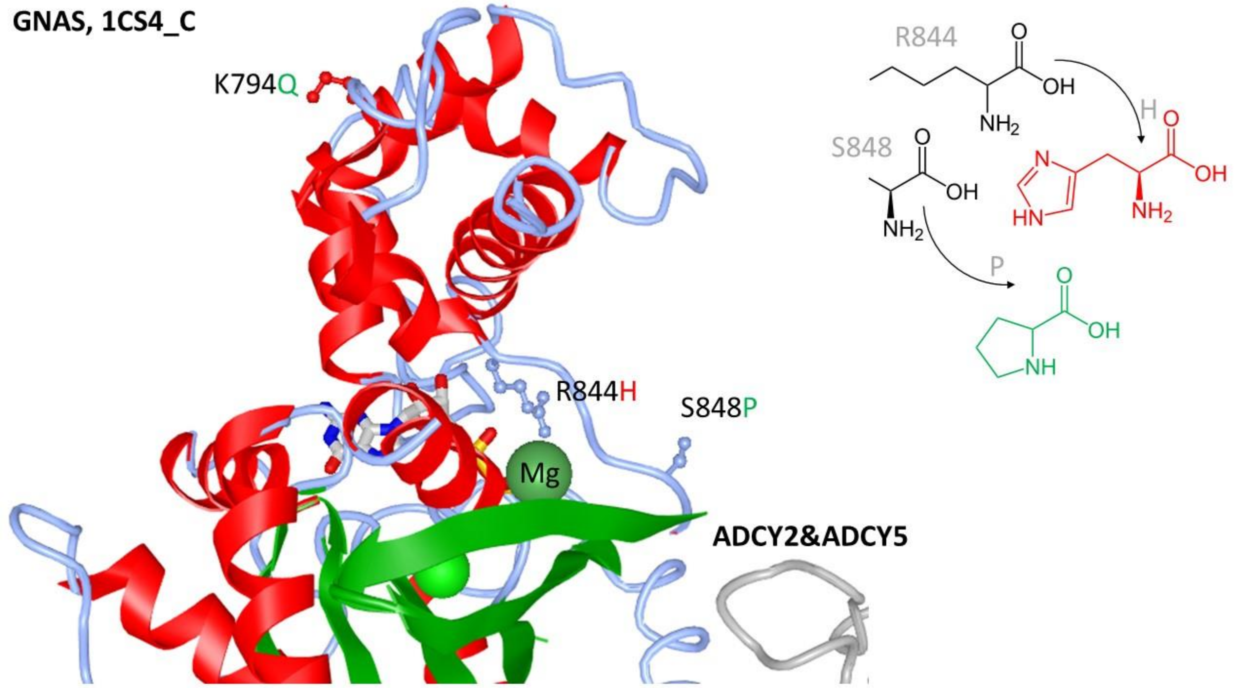

Supplementary Figure 11. Detailed View of *GNAS* Structure

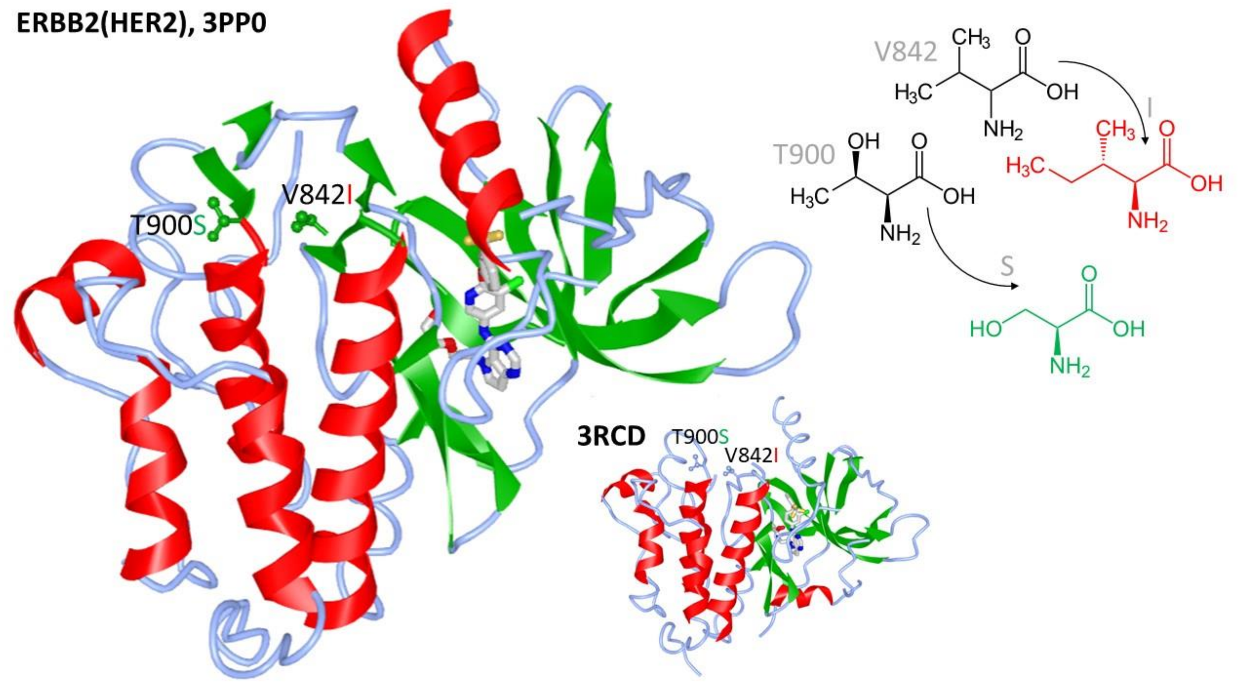

Supplementary Figure 12. Detailed View of *ERBB2* Structure

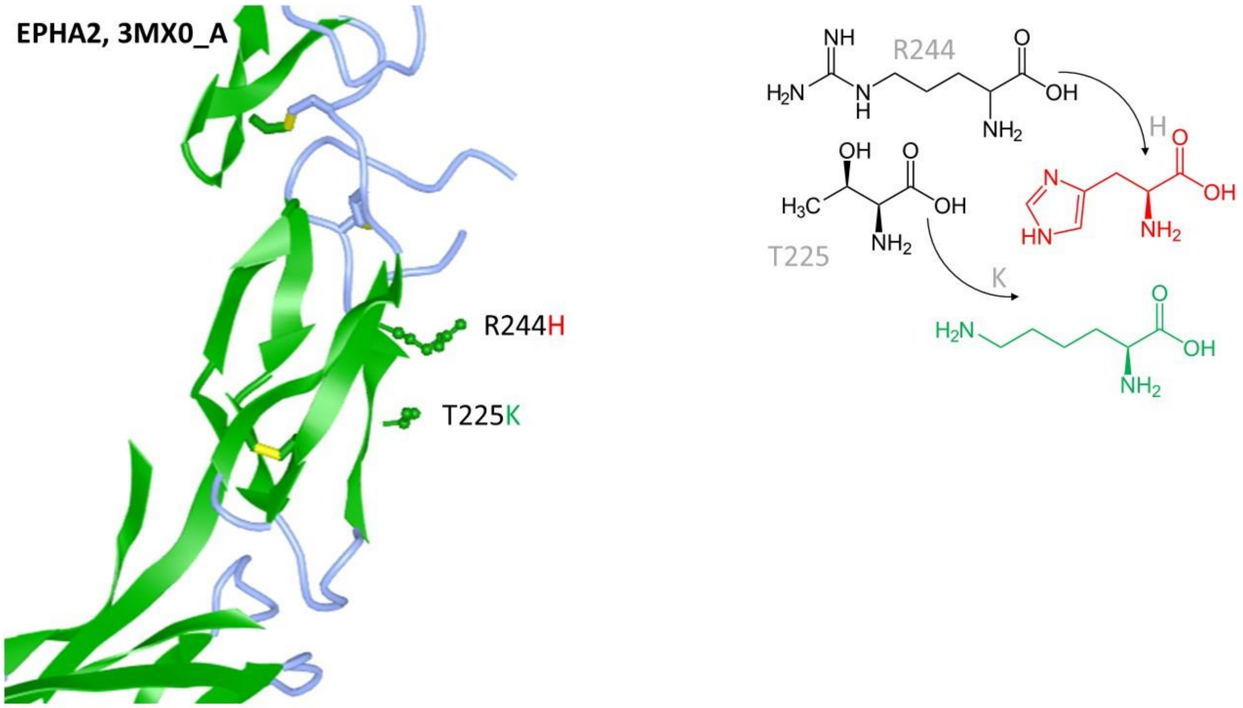

Supplementary Figure 13. Detailed View of *EPHA2* Structure

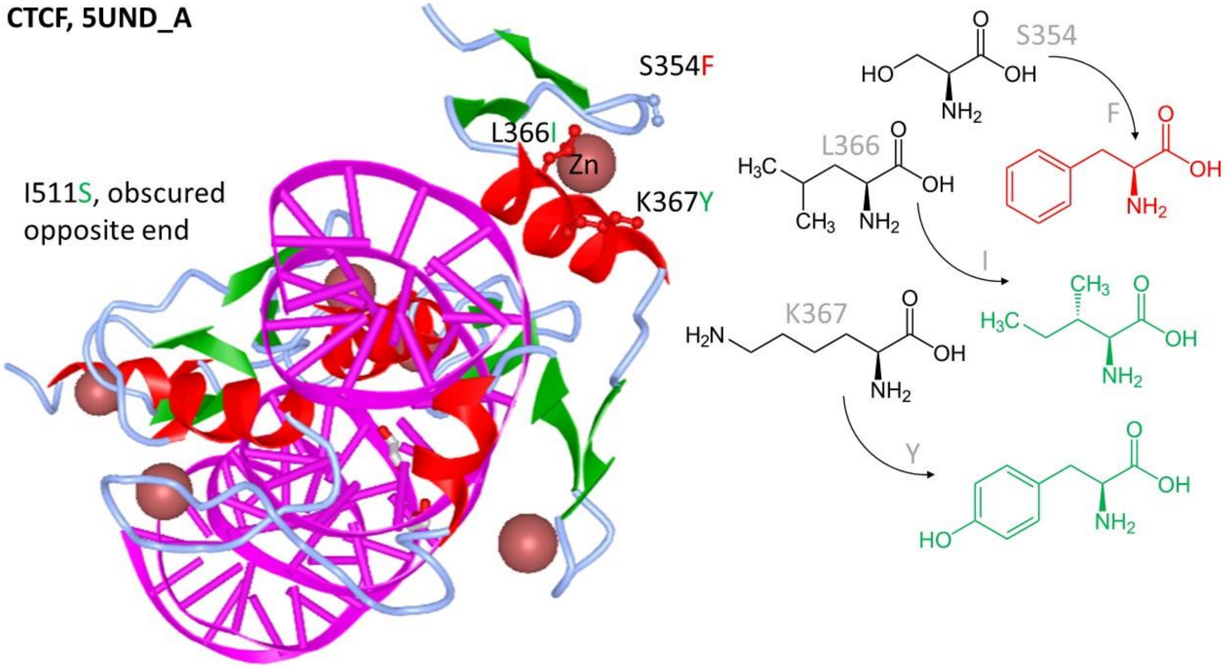

Supplementary Figure 14. Detailed View of *CTCF* Structure

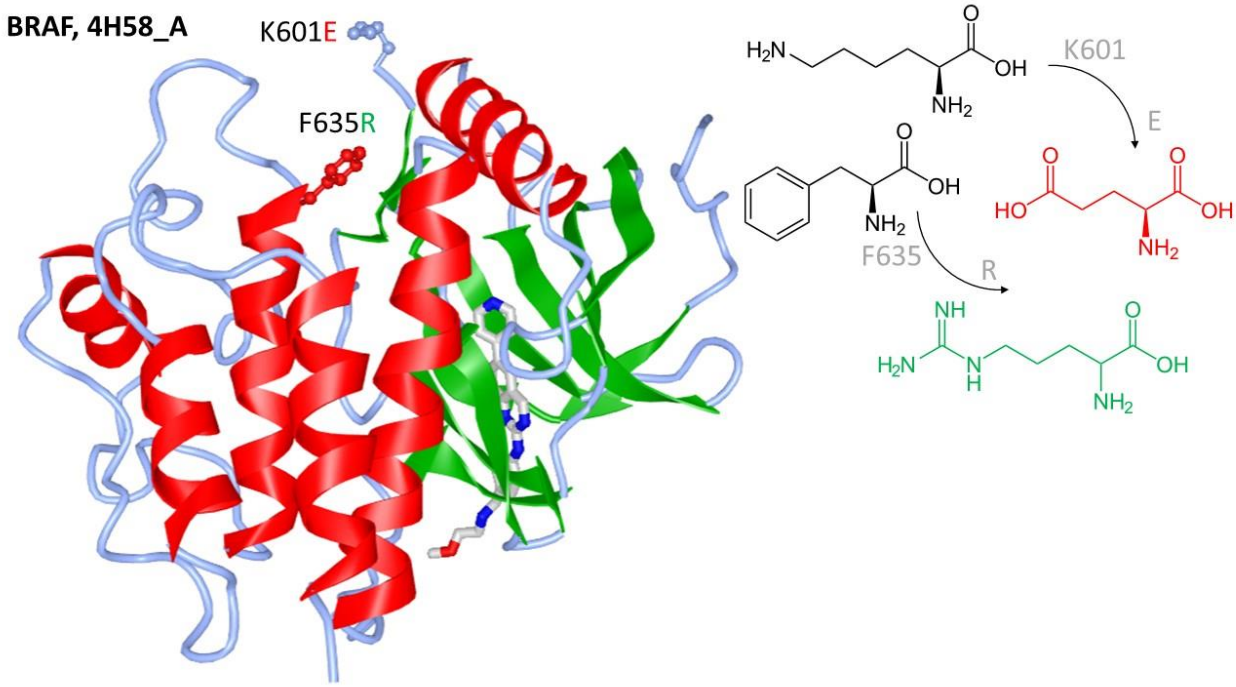

Supplementary Figure 15. Detailed View of *BRAF* Structure

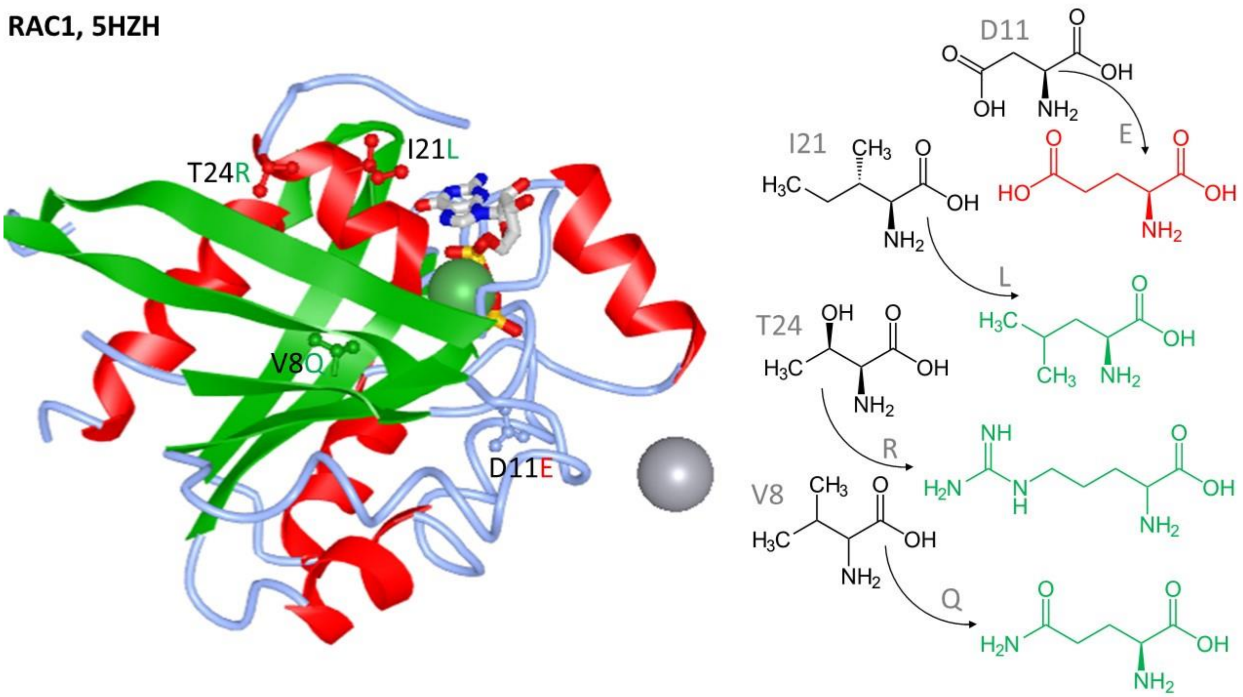

Supplementary Figure 16. Detailed View of *RAC1* Structure

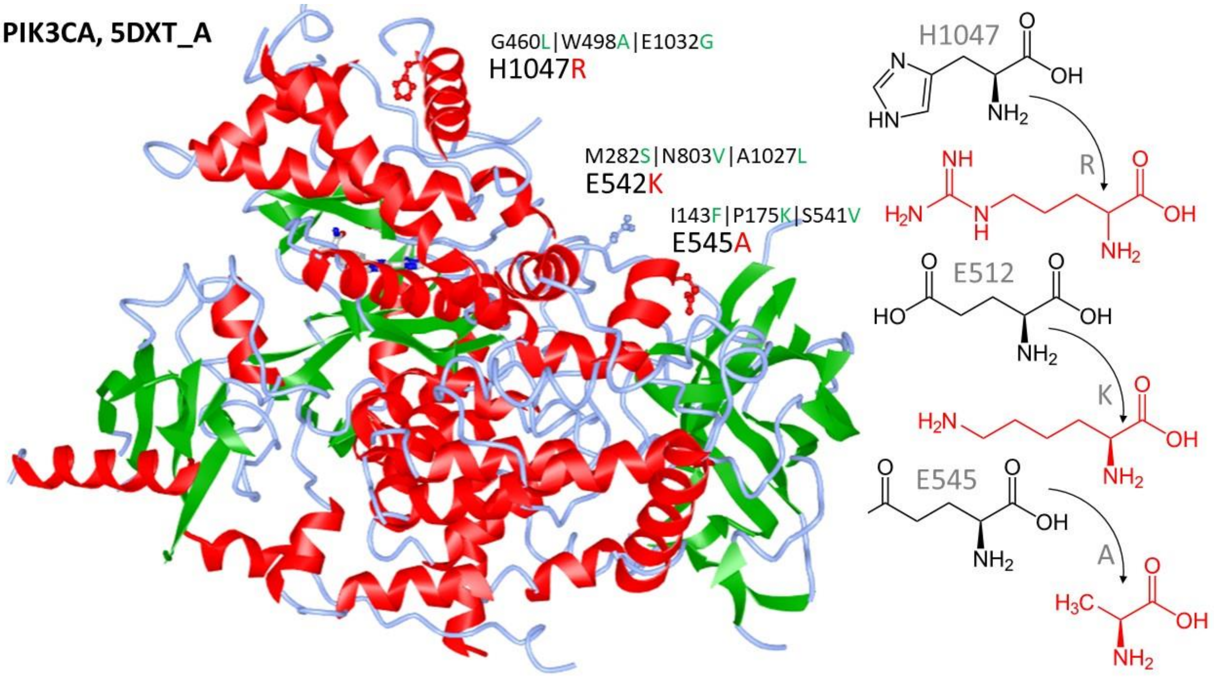

Supplementary Figure 17. Detailed View of *PIK3CA* Structure

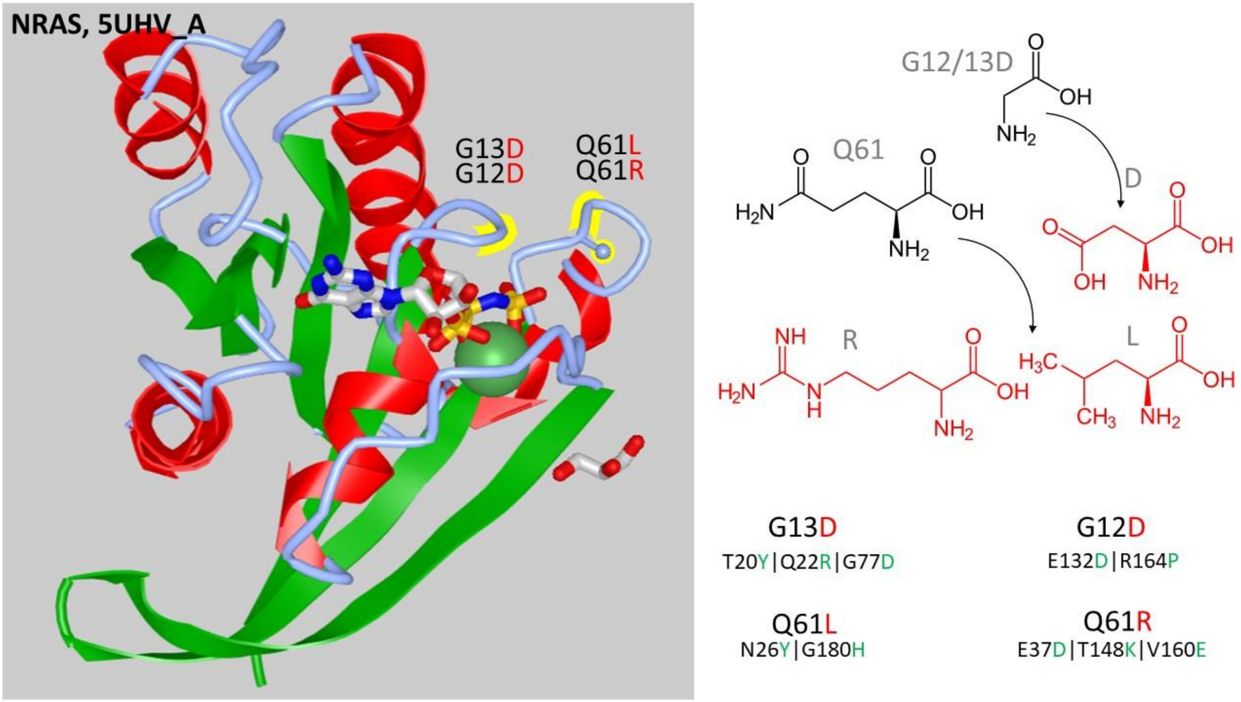

Supplementary Figure 18. Detailed View of *NRAS* Structure

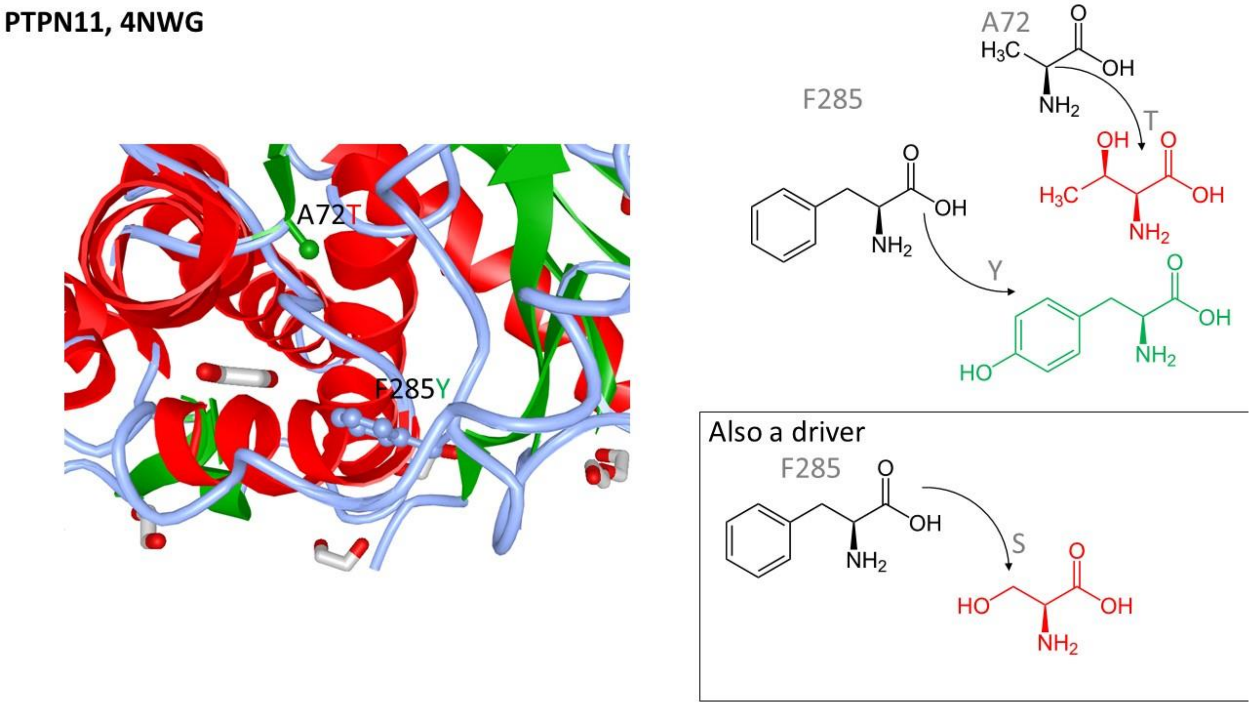

Supplementary Figure 19. Structure of *PTPN11* with E139D mutation, PDB ID: 4NWG

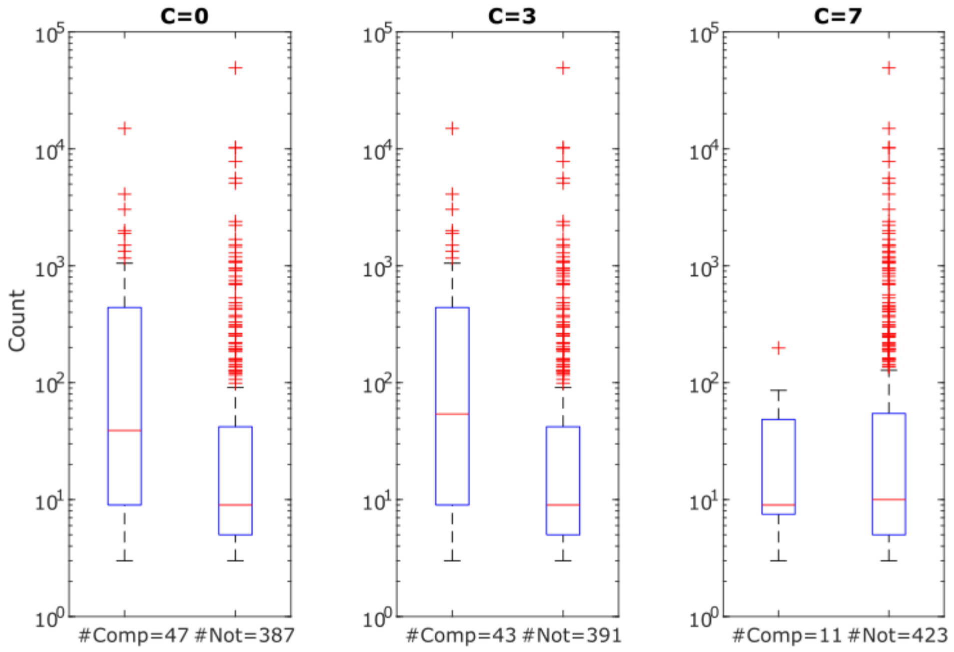

Supplementary Figure 20. Differing Prevalence Among Tumors Between Drivers with Putative Compensators and those Without. For each driver, given a cutoff, C, mutations with high association scores (see Methods: Calculation of Association Score for Pairs of Mutations) paired with that driver among both species and tumors were identified as putative compensators. The 434 drivers considered were then divided into two ensembles based on the identification of at least one putative compensator. Drivers with at least one such mutation identified are more frequently observed (total number of tumors, both compensated and uncompensated) among tumors insensitive to the threshold used. **Note1:** Drivers with a higher overall count are more likely to support evidence of compensation simply because there are greater opportunities for statistically significant associations with a larger number of observations. **Note2:** Many putative compensators are observed in the minority of tumors harboring the associated driver and such a driver is placed in the “compensated” ensemble even if that driver is more often observed “uncompensated”.

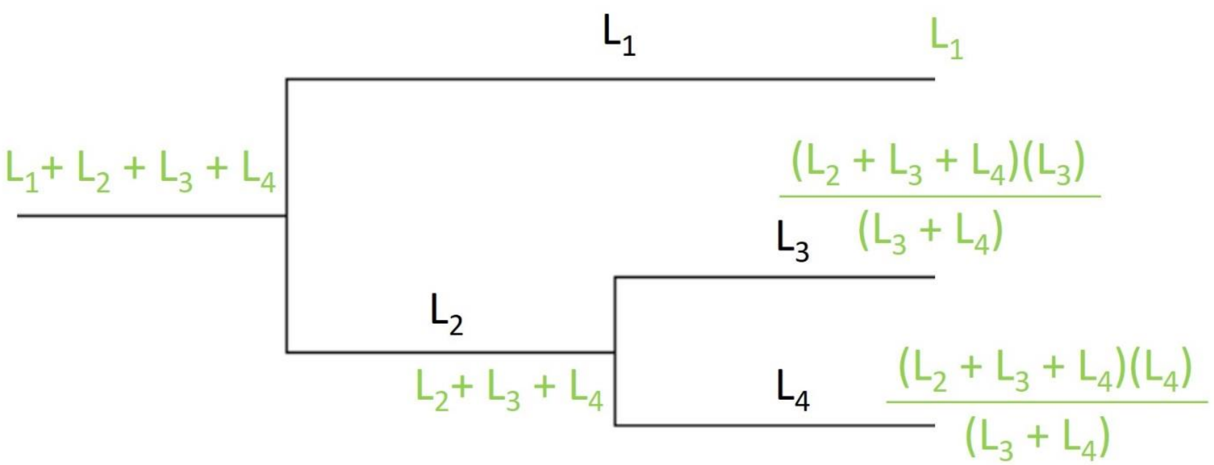

Supplementary Figure 21. Illustration of Leaf-Weighting Procedure Branch lengths are labelled L_i.

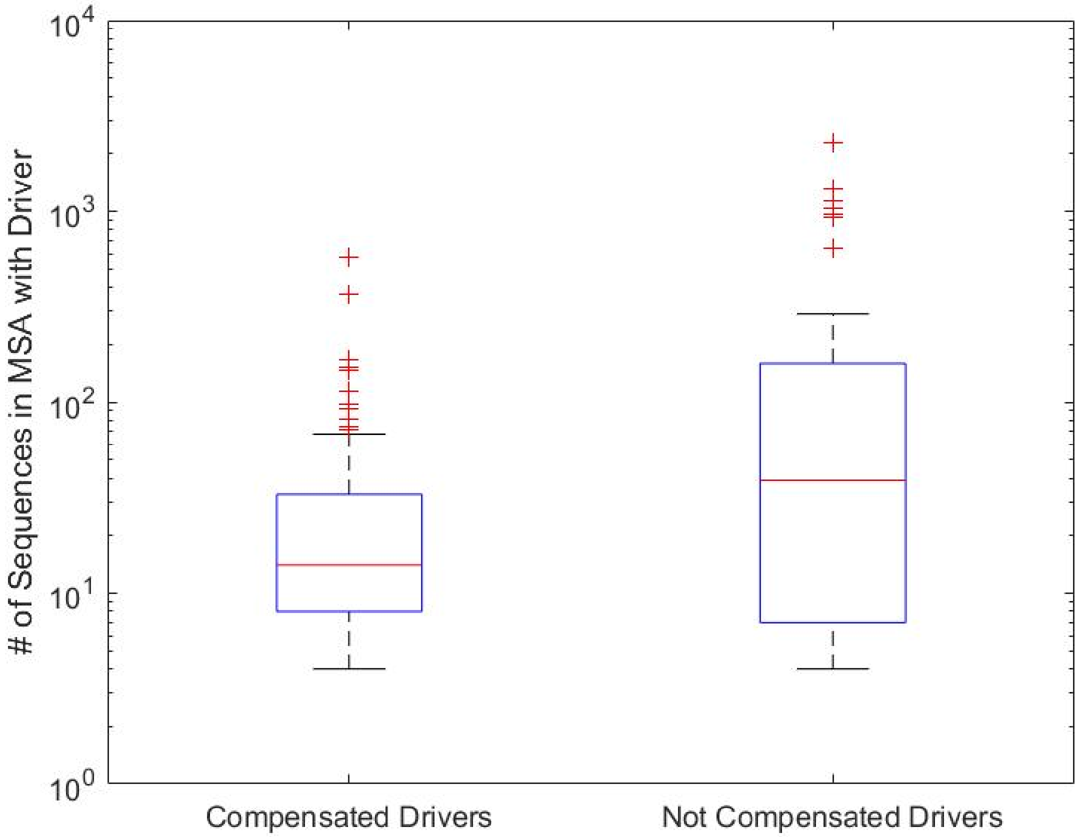

Supplementary Figure 22. Differing Prevalence Among Species Between Drivers with Compensatory Ensembles and those Without All drivers which appear in at least four leaves were divided into two groups: those for which a compensatory ensemble was constructed, and those without. Drivers without a compensatory ensemble tend to be more frequently observed in the MSA than those with a compensatory ensemble.

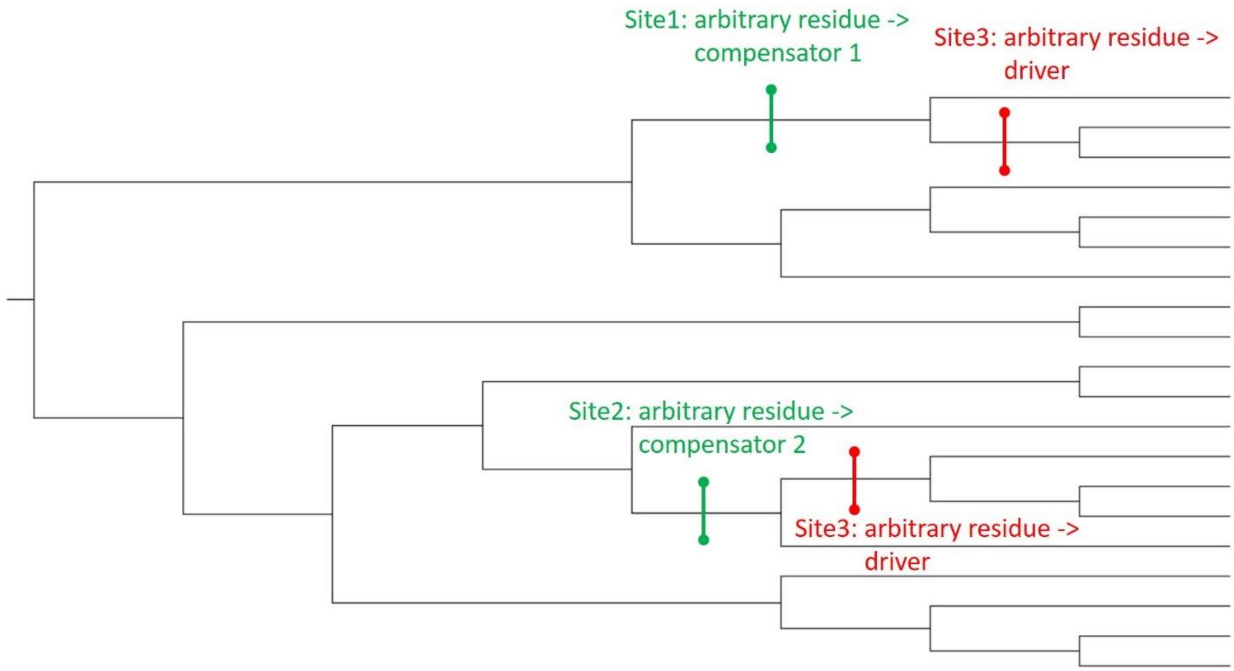

Supplementary Figure 23. Cartoon of Driver/Compensatory Ensemble

References

[1] Rohland, N., & Reich, D. Cost-effective, high-throughput DNA sequencing libraries for multiplexed target capture. *Genome research* ***22*****(5)**, 939-946 (2012).

[2] Forbes, S. A., et al. COSMIC (the Catalogue of Somatic Mutations in Cancer): a resource to investigate acquired mutations in human cancer. *Nucleic acids research* **38(suppl_1)**, D652-D657 (2009).

[3] Weinstein, J. N. et al. The cancer genome atlas pan-cancer analysis project. *Nature genetics* **45(10)**, 1113 (2013).

[4] Bailey, M. H. et al. Comprehensive characterization of cancer driver genes and mutations. *Cell* **173(2)**, 371-385 (2018).

[5] Tamborero, D. et al. Comprehensive identification of mutational cancer driver genes across 12 tumor types. *Scientific reports* **3,** 2650 (2013).

[6] Stratton, M. R., Campbell, P. J., & Futreal, P. A. The cancer genome. *Nature* **458(7239),** 719 (2009).

[7] Iranzo, J., Martincorena, I., & Koonin, E. V. Cancer-mutation network and the number and specificity of driver mutations. *Proceedings of the National Academy of Sciences* **115(26)**, E6010-E6019 (2018).

[8] Fearon, E. R., & Vogelstein, B. A genetic model for colorectal tumorigenesis. *Cell* **61(5)**, 759-767 (1990).

[9] van de Haar, J., Canisius, S., Michael, K. Y., Voest, E. E., Wessels, L. F., & Ideker, T. Identifying Epistasis in Cancer Genomes: A Delicate Affair. *Cell* **177(6)**, 1375-1383 (2019).

[10] Wang, X., Fu, A. Q., McNerney, M. E., & White, K. P. Widespread genetic epistasis among cancer genes. *Nature communications* ***5***, 4828 (2014).

[11] Park, S., & Lehner, B. Cancer type-dependent genetic interactions between cancer driver alterations indicate plasticity of epistasis across cell types. *Molecular systems biology* ***11*****(7)** (2015).

[12] Matlak, D., & Szczurek, E. Epistasis in genomic and survival data of cancer patients. *PLoS computational biology* **13(7)**, e1005626 (2017).

[13] Auslander, N., Wolf, Y. I., & Koonin, E. V. In silico learning of tumor evolution through mutational time series. *Proceedings of the National Academy of Sciences* **116(19)**, 9501-9510 (2019).

[14] Gerstung, M., Eriksson, N., Lin, J., Vogelstein, B., & Beerenwinkel, N. The temporal order of genetic and pathway alterations in tumorigenesis. *PloS one* **6(11)**, e27136 (2011).

[15] Persi, E., Wolf, Y. I., Leiserson, M. D., Koonin, E. V., & Ruppin, E. Criticality in tumor evolution and clinical outcome. *Proceedings of the National Academy of Sciences* **115(47)**, E11101-E11110 (2018).

[16] McFarland, C. D., Korolev, K. S., Kryukov, G. V., Sunyaev, S. R., & Mirny, L. A. Impact of deleterious passenger mutations on cancer progression. *Proceedings of the National Academy of Sciences* **110(8)**, 2910-2915 (2013).

[17] Sharma, P., Hu-Lieskovan, S., Wargo, J. A., & Ribas, A. Primary, adaptive, and acquired resistance to cancer immunotherapy. *Cell* **168(4)**, 707-723 (2017).

[18] Kachalaki, S., Ebrahimi, M., Khosroshahi, L. M., Mohammadinejad, S., & Baradaran, B. Cancer chemoresistance; biochemical and molecular aspects: a brief overview. *European journal of pharmaceutical sciences* **89**, 20-30 (2016).

[19] Sakai, W. et al. Secondary mutations as a mechanism of cisplatin resistance in BRCA2-mutated cancers. *Nature* **451(7182)**, 1116 (2008).

[20] Hirschhorn, R. In vivo reversion to normal of inherited mutations in humans. *Journal of medical genetics* **40(10)**, 721-728 (2003).

[21] Waisfisz, Q. et al. Spontaneous functional correction of homozygous fanconi anaemia alleles reveals novel mechanistic basis for reverse mosaicism. *Nature genetics* **22(4)**, 379 (1999).

[22] Hamanoue, S., Yagasaki, H., Tsuruta, T., Oda, T., Yabe, H., Yabe, M., & Yamashita, T. Myeloid lineage-selective growth of revertant cells in Fanconi anaemia. *British journal of haematology* **132(5)**, 630-635 (2006).

[23] Nikolova, P. V., Wong, K. B., DeDecker, B., Henckel, J., & Fersht, A. R. Mechanism of rescue of common p53 cancer mutations by second-site suppressor mutations. *The EMBO journal* **19(3)**, 370-378 (2000).

[24] Joerger, A. C., Allen, M. D., & Fersht, A. R. Crystal Structure of a Superstable Mutant of Human p53 Core Domain Insights Into The Mechanism Of Rescuing Oncogenic Mutations. *Journal of Biological Chemistry* **279(2)**, 1291-1296 (2004).

[25] Joerger, A. C., Ang, H. C., Veprintsev, D. B., Blair, C. M., & Fersht, A. R. Structures of p53 cancer mutants and mechanism of rescue by second-site suppressor mutations. *Journal of Biological Chemistry* **280(16)**, 16030-16037 (2005).

[26] Baroni, T. E. et al. A global suppressor motif for p53 cancer mutants. *Proceedings of the National Academy of Sciences* **101(14)**, 4930-4935 (2004).

[27] Qutob, N. et al. RGS7 is recurrently mutated in melanoma and promotes migration and invasion of human cancer cells. *Scientific reports* **8(1)**, 653 (2018).

[28] Mateu, M. G., & Fersht, A. R. Mutually compensatory mutations during evolution of the tetramerization domain of tumor suppressor p53 lead to impaired hetero-oligomerization. *Proceedings of the National Academy of Sciences* **96(7)**, 3595-3599 (1999).

[29] Loll, P. J. Membrane proteins, detergents and crystals: what is the state of the art?. *Acta Crystallographica Section F: Structural Biology Communications* ***70*(12)**, 1576-1583 (2014).

[30] Bolla, J. R., Su, C. C., & Yu, E. W. Biomolecular membrane protein crystallization. *Philosophical Magazine* ***92*(19-21)**, 2648-2661 (2012).

[31] Hardy, D., Mandon, E. D., Rothnie, A. J., & Jawhari, A. The yin and yang of solubilization and stabilization for wild-type and full-length membrane protein. *Methods* **147**, 118-125 (2018).

[32] Rogers, M. F., Shihab, H. A., Mort, M., Cooper, D. N., Gaunt, T. R., & Campbell, C. FATHMM-XF: accurate prediction of pathogenic point mutations via extended features. *Bioinformatics*, **34(3)**, 511-513 (2017).

[33] UniProt Consortium. UniProt: a worldwide hub of protein knowledge. *Nucleic acids research*, **47(D1)**, D506-D515 (2018).

[34] Pruitt, K. D., & Maglott, D. R. RefSeq and LocusLink: NCBI gene-centered resources. *Nucleic acids research* **29(1)**, 137-140 (2001).

[35] Fricke, I., & Berken, A. Molecular basis for the substrate specificity of plant guanine nucleotide exchange factors for ROP. *FEBS letters* **583(1)**, 75-80 (2009).

[36] Worthylake, D. K., Rossman, K. L., & Sondek, J. Crystal structure of Rac1 in complex with the guanine nucleotide exchange region of Tiam1. *Nature* **408(6813)**, 682 (2000).

[37] Salgia R. Fibroblast growth factor signaling and inhibition in non-small cell lung cancer and their role in squamous cell tumors. *Cancer medicine* **3(3)**, 681-92 (2014).

[38] Reinhardt, T. & Hinoran, A. Autotracer.org (Raster image to vector conversion platform). https://www.autotracer.org/

[39] Zheng D et al. EGFR G796D mutation mediates resistance to osimertinib. *Oncotarget* **8(30)**, 49671 (2017).

[40] Berman, H.M. et al. The Protein Data Bank. *Nucleic Acids Research* **28(1)**, 235–242 (2000).

[41] Baretić, Domagoj, et al. Structures of closed and open conformations of dimeric human ATM. *Science advances* **3(5)**, e1700933 (2017).

[42] Zhang, B., Zhang, Y., & Shacter, E. Caspase 3-mediated inactivation of rac GTPases promotes drug-induced apoptosis in human lymphoma cells. *Molecular and cellular biology* **23(16)**, 5716-5725 (2003).

[43] Singh, Anju, et al. Dysfunctional KEAP1–NRF2 interaction in non-small-cell lung cancer. *PLoS medicine* **3(10)**, e420 (2006).

[44] Amino Acid Vector Images by NEUROtiker - Own work, Public Domain https://commons.wikimedia.org/w/index.php?curid=1637087

[45] Aertgeerts, Kathleen, et al. Structural analysis of the mechanism of inhibition and allosteric activation of the kinase domain of HER2 protein. *Journal of Biological Chemistry* **286(21)**, 18756-18765 (2011).

[46] Ishikawa, Tomoyasu, et al. Design and synthesis of novel human epidermal growth factor receptor 2 (HER2)/epidermal growth factor receptor (EGFR) dual inhibitors bearing a pyrrolo [3, 2-d] pyrimidine scaffold. *Journal of medicinal chemistry* **54(23)**, 8030-8050 (2011).

[47] Vasbinder, Melissa M., et al. Discovery and optimization of a novel series of potent mutant B-RafV600E selective kinase inhibitors. *Journal of medicinal chemistry* **56(5)**, 1996-2015 (2013).

[48] Himanen, Juha P., et al. Architecture of Eph receptor clusters. *Proceedings of the National Academy of Sciences* **107(24)**, 10860-10865 (2010).

[49] Johnson, Christian W., et al. The small GTPases K-Ras, N-Ras, and H-Ras have distinct biochemical properties determined by allosteric effects. *Journal of Biological Chemistry* **292(31)** 12981-12993 (2017).

[50] dal Maso, Emma, et al. The Molecular Control of Calcitonin Receptor Signaling. *ACS Pharmacology & Translational Science* **2(1)**, 31-51 (2019).

[51] Tesmer, John JG, et al. Molecular basis for P-site inhibition of adenylyl cyclase. *Biochemistry* **39(47)**, 14464-14471 (2000).

[52] Dagliyan, Onur, et al. Engineering extrinsic disorder to control protein activity in living cells. *Science* **354(6318)**, 1441-1444 (2016).

[53] Sogabe, Satoshi, et al. Discovery of a Kelch-like ECH-associated protein 1-inhibitory tetrapeptide and its structural characterization. *Biochemical and biophysical research communications* **486(3)**, 620-625 (2017).

[54] Liu, X., Coker, S., Ceska, T., Baker, T. De novo design and crystallographic validation of antibodies targeting a pre-selected epitope. To be published.

[55] Hashimoto, Hideharu, et al. Structural basis for the versatile and methylation-dependent binding of CTCF to DNA. *Molecular cell* **66(5)**, 711-720 (2017).

[56] Heffron, Timothy P., et al. The Rational Design of Selective Benzoxazepin Inhibitors of the α-Isoform of Phosphoinositide 3-Kinase Culminating in the Identification of (S)-2-((2-(1-Isopropyl-1 H-1, 2, 4-triazol-5-yl)-5, 6-dihydrobenzo [f] imidazo [1, 2-d][1, 4] oxazepin-9-yl) oxy) propanamide (GDC-0326). *Journal of medicinal chemistry* **59(3)**, 985-1002 (2016).

[57] Kops, G. J., Weaver, B. A., & Cleveland, D. W. On the road to cancer: aneuploidy and the mitotic checkpoint. *Nature Reviews Cancer* **5(10)**, 773-785 (2005).

[58] Ben-David, Uri, and Angelika Amon. Context is everything: aneuploidy in cancer. *Nature Reviews Genetics* 1-19 (2019).

[59] Chen, W., Li, Y., & Wang, Z. Evolution of oncogenic signatures of mutation hotspots in tyrosine kinases supports the atavistic hypothesis of cancer. *Scientific reports* **8(1)**, 1-8 (2018).

[60] Gültas, M., Haubrock, M., Tüysüz, N., & Waack, S. Coupled mutation finder: A new entropy-based method quantifying phylogenetic noise for the detection of compensatory mutations. *BMC bioinformatics* **13(1)** 225 (2012).

[61] Hopf, T. A. et al. The EVcouplings Python framework for coevolutionary sequence analysis. *Bioinformatics* **35(9)** 1582-1584 (2019).

[62] Stiffler, M. A. et al. Protein structure from experimental evolution. *Cell Systems* (2019).

[63] Qiu, W. et al. To Be Published

[64] Malhis, N., Jones, S. J., & Gsponer, J. Improved measures for evolutionary conservation that exploit taxonomy distances. *Nature communications* **10(1)**, 1-8 (2019).

[65] Breen, M. S., Kemena, C., Vlasov, P. K., Notredame, C., & Kondrashov, F. A. Epistasis as the primary factor in molecular evolution. *Nature* **490(7421)**, 535 (2012).

[66] Kondrashov, A. S., Sunyaev, S., & Kondrashov, F. A. Dobzhansky–Muller incompatibilities in protein evolution. *Proceedings of the National Academy of Sciences* **99(23)**, 14878-14883 (2002).

[67] Gao, L., & Zhang, J. Why are some human disease-associated mutations fixed in mice? *Trends in Genetics* **19(12)**, 678-681 (2003).

[68] Poon, A., Davis, B. H., & Chao, L. The coupon collector and the suppressor mutation: estimating the number of compensatory mutations by maximum likelihood. *Genetics* **170(3)**, 1323-1332 (2005).

[69] Camps, M., Herman, A., Loh, E. R. N., & Loeb, L. A. Genetic constraints on protein evolution. *Critical reviews in biochemistry and molecular biology* **42(5)**, 313-326 (2007).

[70] Altschul, Stephen F., et al. Gapped BLAST and PSI-BLAST: a new generation of protein database search programs. *Nucleic acids research* **25(17)**, 3389-3402 (1997).

[71] Wolf, Yuri I., et al. Origins and evolution of the global RNA virome. *MBio* **9(6)**, e02329-18 (2018).

[72] Price, Morgan N., Paramvir S. Dehal, and Adam P. Arkin. FastTree 2–approximately maximum-likelihood trees for large alignments. *PloS one* **5(3)**, e9490 (2010).

[71] Yutin, Natalya, et al. The deep archaeal roots of eukaryotes. *Molecular biology and evolution* **25(8)**, 1619-1630 (2008).

[74] Makarova, Kira, Yuri Wolf, and Eugene Koonin. Archaeal clusters of orthologous genes (arCOGs): an update and application for analysis of shared features between Thermococcales, Methanococcales, and Methanobacteriales. *Life* **5(1)**, 818-840 (2015).

[75] Fitch, Walter M. Toward defining the course of evolution: minimum change for a specific tree topology. *Systematic Biology* **20(4)**, 406-416 (1971).
