# Supplementary material for: Deep phylogeny of cancer drivers and compensatory mutations
Source: Commun Biol. 2020 Oct 2;3:551. doi: 10.1038/s42003-020-01276-7 (PMC7532533; doi:10.1038/s42003-020-01276-7)
Supplement: Supplementary file 2 — Description of Additional Supplementary Files [file 42003_2020_1276_MOESM2_ESM.pdf]

Description of Additional Supplementary Files

File Name: Supplementary Data 1
Description:
Name= “GENE|RefseqID”
Newick(leaves)= “Reduced_Taxonomy_|RefseqID”

Newick tree files with leaves labelled as follows are made available in tab delimited format with two fields, “Name” and “Newick”. Supplementary Data 1 contains one tree for each of the 84 driver genes studied in this work.

File Name: Supplementary Data 2
Description: drivers present in 3 or more leaves
Excel file containing relevant structure, tree presentation, and literature references.

File Name: Supplementary Data 3
Description: drivers present in 3 or more leaves
Text file containing reduced alignments of i) all human sequences present in the MSA; ii) representative sequences across the phylogenetic depth of the MSA; and iii) representative sequences harboring the mutation(s) of interest; a list of the most frequent mutations in COSMIC; and a logical representation of their status as a recognized common human polymorphism:

File Name: Supplementary Data 4
Description:
Name= “GENE|RefseqID|DriverAA”
Newick(leaves)= “Reduced_Taxonomy_|_Driver.Index_|RefseqID”
Newick tree files with leaves labelled as follows are made available in tab delimited format with two fields, “Name” and “Newick”. Supplementary Data 4 contains one tree for each driver present in 3 or more leaves. Each driver is labelled Driver.Index. The drivers are indexed over the number of times a transition to the driver appears in the tree.

File Name: Supplementary Data 5
Description: all “associated pairs” in the MSA with a driver
Excel file containing relevant structure, tree presentation, and literature references.

File Name: Supplementary Data 6
Description: all “associated pairs” in the MSA with a driver
Text file containing reduced alignments of i) all human sequences present in the MSA; ii) representative sequences across the phylogenetic depth of the MSA; and iii) representative sequences harboring the mutation(s) of interest; a list of the most frequent mutations in COSMIC; and a logical representation of their status as a recognized common human polymorphism:

File Name: Supplementary Data 7
Description: all “associated pairs” in the COSMIC database with a driver

Excel file containing relevant structure, tree presentation, and literature references.

File Name: Supplementary Data 8
Description: all “associated pairs” in the COSMIC database with a driver
Text file containing reduced alignments of i) all human sequences present in the MSA; ii) representative sequences across the phylogenetic depth of the MSA; and iii) representative sequences harboring the mutation(s) of interest; a list of the most frequent mutations in COSMIC; and a logical representation of their status as a recognized common human polymorphism:

File Name: Supplementary Data 9
Description: outlier “associated pairs” in both the MSA and COSMIC
Excel file containing relevant structure, tree presentation, and literature references.

File Name: Supplementary Data 10
Description: outlier “associated pairs” in both the MSA and COSMIC
Text file containing reduced alignments of i) all human sequences present in the MSA; ii) representative sequences across the phylogenetic depth of the MSA; and iii) representative sequences harboring the mutation(s) of interest; a list of the most frequent mutations in COSMIC; and a logical representation of their status as a recognized common human polymorphism:

File Name: Supplementary Data 11
Description: compensators which have a mutation in the same site in COSMIC
Excel file containing relevant structure, tree presentation, and literature references.
5

File Name: Supplementary Data 12
Description: all compensatory ensembles
Excel file containing relevant structure, tree presentation, and literature references.

File Name: Supplementary Data 13
Description: all compensatory ensembles
Text file containing reduced alignments of i) all human sequences present in the MSA; ii) representative sequences across the phylogenetic depth of the MSA; and iii) representative sequences harboring the mutation(s) of interest; a list of the most frequent mutations in COSMIC; and a logical representation of their status as a recognized common human polymorphism:

File Name: Supplementary Data 14
Description:
Name= “GENE|RefseqID|DriverAA|Compensatory_Ensemble_AA”
Newick(leaves)= “Reduced_Taxonomy_|_Driver.Index_|_t1.Index1.Index2_|RefseqID"
Newick tree files with leaves labelled as follows are made available in tab delimited format with two fields, “Name” and “Newick”. Supplementary Data 14 contains one tree for each compensatory ensemble. Each compensatory mutation is labelled t1.Index1.Index2 (“t1” because the transition to the compensatory mutation appears

ancestral to the “t2” driver). The drivers are indexed over the number of times a transition to the driver appears in the tree. Index1 specifies the member of the compensatory ensemble and Index2 specifies the transition to that compensatory mutation. “FALSE” appears in place of the driver index or Index2 for leaves in these labelled clades which do not harbor the state in the extant sequence. Transitions to every driver are labelled; however, only transitions to compensators which are ancestral to drivers are labelled and in many cases, there are many unlabeled compensators in the tree. In cases where a leaf shares multiple labelled ancestral states, the leave label reflects all labels.

File Name: Supplementary Data 15
Description: symbol, name, accession code
Text file containing the gene symbol, gene name, and protein accession code.
